# Direct imaging of capillaries reveals the mechanism of arteriovenous interlacing in the chick chorioallantoic membrane

**DOI:** 10.1038/s42003-018-0229-x

**Published:** 2018-12-21

**Authors:** Sophie Richard, Amanda Brun, Antonio Tedesco, Benjamin Gallois, Naoual Taghi, Philippe Dantan, Johanne Seguin, Vincent Fleury

**Affiliations:** 1 Laboratoire Matière et Systèmes Complexes, Université Paris Diderot/CNRS UMR 7057, 10 rue Alice Domont et Léonie Duquet, 75013 Paris, France; 2 Department of Chemistry, São Paulo University-FFCLRP, Center of Nanotechnology and Tissue Engineering-Photobiology and Photomedicine Research Group, Av. Bandeirantes 3900, Ribeirão Preto, SP 14040-901 Brazil; 3 Unité de Technologies Chimiques et Biologiques pour la Santé (UTCBS), Université Paris Descartes/CNRS UMR 8258/Inserm U1022, 4 avenue de l’Observatoire, 75006 Paris, France

**Keywords:** Cardiovascular biology, Angiogenesis, Time-lapse imaging

## Abstract

Understanding vascular development in vertebrates is an important scientific endeavor. Normal vasculatures generally start off as a disorganized capillary lattice which progressively matures into a well-organized vascular loop comprising a hierarchy of arteries and veins. One striking feature of vascular development is the interlacing of arteries and veins. How arteries and veins manage to avoid themselves and interlace with such a perfect architecture is not understood. Here we present a detailed view of the development of the vasculature in the chorioallantoic membrane of the chicken embryo. We find that the origin of arteriovenous interlacing lies in the presence of an increased hemodynamic resistance at the distal part of the arteries due to vascular flattening onto the ectodermal surface. This reduces the vascular conductance distally, thus repelling veins away. In more proximal parts, vessels round off into cylinders and the increased flow attracts veins.

## Abstract (web summary)

Sophie Richard et al. present a detailed study of the development of the vasculature in the chicken embryo chorioallantoic membrane. They find that the interlacing of arteries and veins arises due to differential hemodynamic resistance acting on the distal and proximal ends of the arteries causing veins to be alternately repelled or attracted.

## Introduction

Vascular pathologies are a major cause of death in Western countries^1,2^. Malignant tumors develop by de novo stimulation of vascular development^3^, which makes neoangiogenesis an interesting target for therapy. Understanding vascular development is therefore very important. Since the pioneering work of Thoma^4^ (reviewed by Hugues^5^), the chicken embryo has been used to investigate fundamental processes in vascular development. The formation of a functional vasculature is a highly dynamic phenomenon^5–8^ which has been amply documented^9–17^. It takes a few days in model systems such as the chicken yolk-sac or chorioallantoïc membrane (CAM). It is a multiscale process with rapid (on the order of hours) developmental changes from the capillary level up to the main vessels^18^. Hemodynamic forces feedback to remodel the vessels^5,19^, giving ground to self-organization principles such as Murray’s laws^20–22^ or Zamir’s law^23^. These laws are based on physical principles and are therefore expected to be independent of a specific system or animal species^24^. Recently, the effects of the pulsatile character of the flow due to the heartbeat^25^ or of the slow viscoelastic creep of the tissue^26^ have been taken into account.

The chicken is widely used because the extraembryonic organs can be accessed through a window in the shell, or via shell-less experimentation^9,27,28^. One distinct feature of vascular patterns in this animal model is the interdigitation of arteries (A) and veins (V). In organs such as the CAM, it is generally observed that arteries and veins avoid each other by interdigitating. This dense interlacing with narrow approach of arteries and veins is crucial for proper blood flow across capillaries and normal tissue oxygenation^11^. Conversely, direct A-V connections (shunts or fistulae) are detrimental because they prevent blood flow through the capillaries, thereby strongly reducing gas exchanges (O_2_, CO_2_). Brain micro-vascular shunts are for example observed in the pathogenesis of high intracranial pressure^29^. The interdigitating structure is present in the CAM and in the human or simian retinas^24^ CAM vessels interdigitate at all scales and present a measurable deviation from Murray’s law^11^. This suggests that other formation principles might be at play. In particular, the origin of vascular interlacing, which is one of the most striking features of vascular patterns, is not understood.

We have developed an automatic image processing method which allows one to extract the structure of the capillaries, and of all vessels at higher levels in the spatial hierarchy by direct in vivo optical imaging without fluorescence, fixation, cast or staining (see Methods, Optical Imaging). The technique we developed represents a major step forward compared to previous imaging methods^11,13–17^ (See especially the Table in the appendix of ref. ^13^ “Overview of the important studies in which pre- and postcapillary blood vessels in the CAM were analyzed”). We use erythrocytes as point-like tracers of the vessels, and integrate their position over time. The main problems in capillary imaging are the intrinsic movements of the embryo (spontaneous skeletal muscle contraction, heartbeat and tissue peristaltic movements). In brief, our method^30^ consists of acquiring a large number of images (50 < N < 300) and performing sequential steps of decorrelation, elimination of blurred frames, registration, and stack summing or averaging. This process yields two distinct images of the vasculature: the image of the vasculature itself (the lumina), and the image of the perfusion. Our current algorithm is able to automatically generate time-lapse movies of tissue perfusion with a 30-sec frame interval. (In ref. ^31^ a similar technique might have been used to generate static images, however, these authors do not provide details of the acquisition method nor of its performances).

The study presented here focuses on days 7–13 of chicken development. CAMs were imaged between day 5 (onset of CAM formation) and day 14, but most analyses were performed between day 8 and 13. The rounded shape of the sac, and the vicinity of the embryo complicate imaging at earlier stages. As from ~7 days, a wide part of the CAM is flattened against the top surface of the yolk, far from the embryo. The vasculature remains roughly in the same plane and bulk displacement is therefore small. Beyond 13 days, we observed slow chronic contractions of the CAM probably related to smooth muscle cells around arterioles, or to the contractility of extraembryonic organs^5,32,33^. This contraction causes arteries to stretch while veins become varicose. This effect deserves a separate study.

In this report, we use our new imaging technique to address the long-standing question of artero-venous (A-V) interlacing. The work presented here shows that the tips of arterioles are flattened, thereby increasing hemodynamic resistance in the distal parts of the arteries. Further away from the tips, more proximally, vessels remain cylindrical. Because veins are sensitive to shear^4–6^, they are attracted hemodynamically towards the proximal regions where blood flow is higher. Tip flattening and the associated tissue swelling act as a short-range repulsive force between tips of arteries and veins. This provides a physical mechanism for A-V interlacing.

## Results

### Presence in arteries of a flat terminal segment

**Fig. 1:**
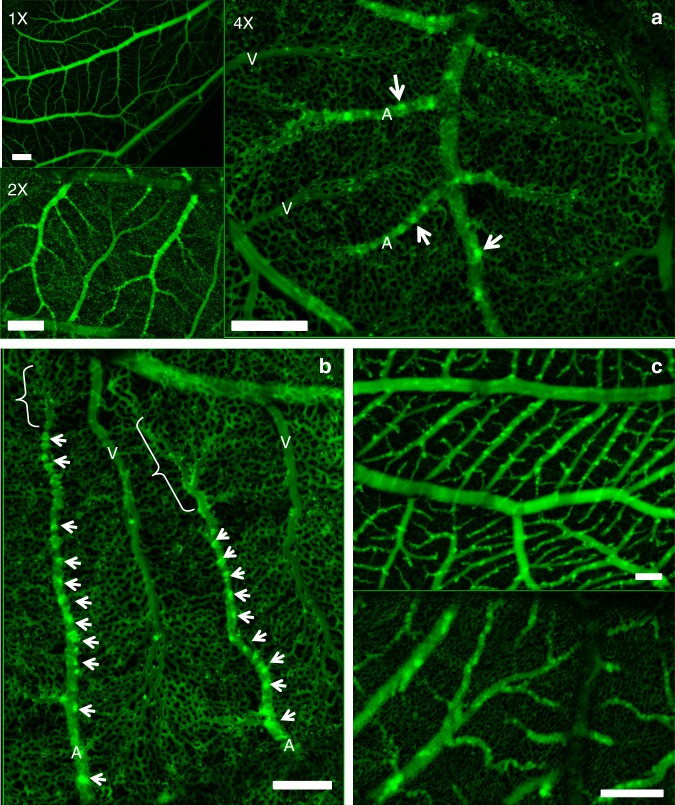
Images obtained with our technique, of a CAM vasculature at day 10 of development. The images are taken with a low resolution binocular, which allows one to have a wide field (×1) and still be able to zoom in at the level of capillaries (×4). The interlaced structure is quite obvious. Arteries and veins are identified by the movie of the flow, but also by conspicuous differences in branching pattern, and in the form of neighboring capillaries (see. Figure 2). **a** Scale bar 250 μm. **b** Scale bar 250 μm. **b** shows the terminal arterioles. It is observed that there exist vertical chimneys, regularly spaced along the arterioles, from which the flow is higher (and not along veins). These chimneys are visible by hotspots (arrows) corresponding to erythrocytes flowing towards the reader. They appear bright because of the stacking effect of the red cells which increases the contrast. However, as one approaches the distal end of the arteriole, there exists a flat area devoid of vertical chimneys (brackets in Fig. 1b). **c** Scale bar 200 μm.The hotspots pointing vertically in 3D are present constantly, up to the 14th day of development, which was the terminal stage of this study. As the CAM develops, the interlacing becomes thinner, with smaller capillary anastomoses. This tendency of the vascular pattern to become more refined with thinner anastomoses has been reported before^16^. Such images as **c** are more difficult to obtain considering the smaller size of the capillaries, and the ample embryo movements at this stage. Still, we were able to image the capillary lattice, and the vessels at the upper levels of the hierarchy, by filming the embryo at the periphery of the plastic cup where movements are smaller. It is noteworthy that vessels begin to meander. This meandering of the vessels was actually related to slow contraction of the entire CAM which buckles the veins (the slow CAM contraction is already visible by the end of Supplementary Movie 1)

**Fig. 2:**
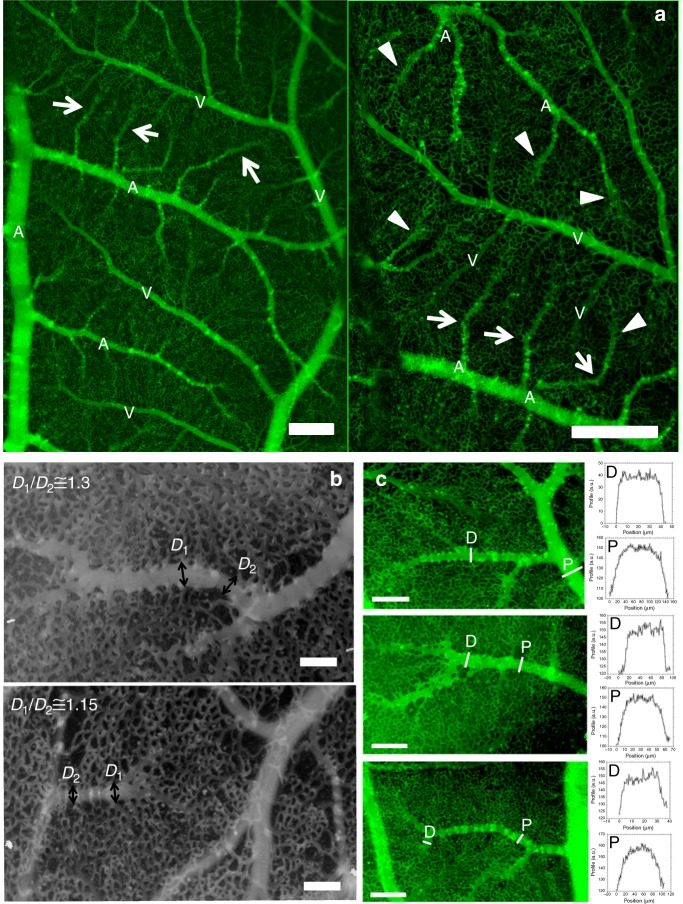
Vascular structure of the CAM during interlacing. **a**, left panel: At low resolution, all arterioles exhibit a terminal segment which is distinctly different from the proximal segment (arrows). The arterial pattern shows the presence of vertical “chimneys” or “springs” of flow identified by the presence of “hotspots” along the arterioles. The hotspots provide flow sources located proximally with respect to the arteriolar hierarchy. The veins do not “navigate” towards the tip of arteries where the flat distal areas are found. **a**, right panel: Magnification shows that the flat distal areas are wider than the more proximal part of the arteries. The distal segment often has a “goose leg” widening (arrowheads). Scale bar 250 μm. **b** Scale bar 100 μm. One can in many cases identify the exact point of contact between the 3D arteriole and the ectoderm (Supplementary Movie 2). There, the vessel spreads, and an increase of diameter of order 15–30% is observed (**b** top panel is a day 11 embryo, bottom is a day 13). The flattened segments of the arteries tend to have regularly spaced sawtooth collaterals. The interspace distance between flat collaterals is the same as the interspace distance of the vertical chimneys as seen in (**b**) (bottom), and also in (**a**). **c** Scale bar 150 μm. When plotting the optical profile one finds that the terminal parts of the arterioles appear flat (top graphs for each arteriole), while more proximal vessels appear round (bottom graphs). The white lines correspond to the locations where the profiles were extracted, D stands for distal and P for proximal (see also Supplementary Information). This shows that the terminal part of the arteriole is flattened on the underneath surface of the ectoderm on which it creeps (*N* = 30 arterioles were processed giving similar results)

Our method reveals in Fig. 1a the structure of the vasculature, during interlacing (day 10) in the CAM (Fig. 1a and Supplementary Fig. 1). A time-lapse of development of the CAM vasculature between day 10 and 13 is shown in Supplementary Movie 1. Although very thin at day 10, the CAM has a stratified 3D structure. Arteries and veins are segregated in different planes, with veins on top and arteries beneath them^9^. Vertical tubes (“chimneys”) form in the more proximal part (upstream, Fig. 1b arrowheads). They appear as optically dense spots along the blood vessels. These dense spots are visible at all stages after day 8 (Fig. 1c). These vertical vessels have a greater diameter than the surrounding capillaries. Chimneys are absent in the most distal part of the arterioles (Fig. 2a, arrows). Strikingly, the terminal segment of arterioles is 20% wider (Fig. 2b, and Supplementary Movie 2) than its more proximal part. (Fig. 2a, arrowheads). It has a bilateral “sawtooth” pattern with regularly spaced in-plane collaterals (Fig. 2a, arrows). The terminal part of the vessel displays a flat profile (Fig. 2c), while more mature, proximal vessels (with vertical chimneys) have a rounder profile. The terminal segments of the arteries do not form cylindrical tubes: they are flattened against the ectodermal surface.

### Presence around the terminal segment of a swollen area

**Fig. 3:**
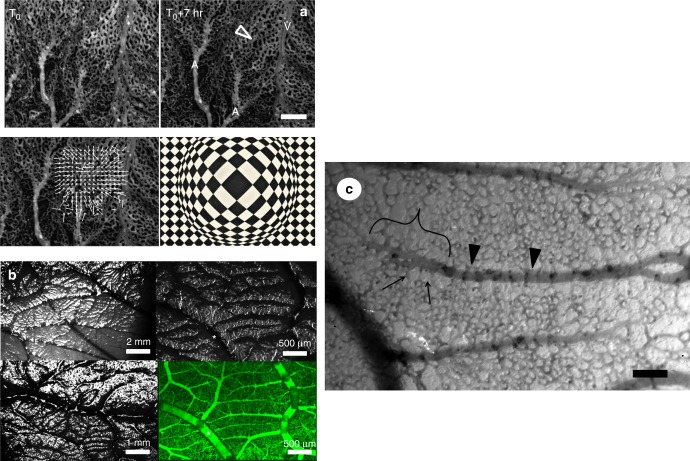
Observation of tissue dilation. **a** Scale bar 250 μm. Progression of a “flat” arteriole is accompanied by a dilation of the tissue underneath and surrounding it (PIV tracking in the bottom left). It is observed that the capillary anastomoses widen (arrowhead in top right), in a pattern similar to a “Vasarely” painting. The central anastomoses widen while the peripheral widen less, and the venous, squeezed, path actually narrows. **b**. Shadowgraphic inspection of the surface of a day 13 embryo (see Supplementary Figure 2 for the setup), reveals a pattern of micro-swellings fitting exactly the vascular pattern. The white flee in the images is positioned at the same place. **c** Scale bar 100 μm. When the vascular pattern can be imaged together with the surrounding cells (this is possible after day ~11 when part of the CAM floats on top of the albumin, instead of the yolk), one observes that cells are dilated along and on top of the vessels, while they are smaller away from the vessels. In the distal part of an arteriole (bracket), the swelling is below the vessels, while more proximally the swelled cells cross the vessel (arrowheads), as the vessels delaminate. The distal parts are thus flattened against the ectoderm, while proximally the vessels round off, and the collaterals rock vertically. In the flattened area, the collateral interspace is fixed by the size of swollen cells (arrows)

Flow visualization (Supplementary Movie 3, Mag. ×4  ) gives the impression that the venous flow swerves around a denser or thicker area, as if the arterial bed was swollen exactly where the terminal segments are flattened. When the processed images are followed in Time-Lapse (Supplementary Movie 4), the denser/thicker areas appear more rigid as the surrounding tissue (the distal part moves en bloc, with its surrounding capillaries, this also explains the kinks in the vessel of Fig. 2a). The onset of inflation in these areas (Supplementary Movies 5, 6) shows that swelling repels veins by two mechanisms: physical displacement, like “hoses” of tubes (a tube is physically displaced sideways), and/or a poro-elastic shift of active capillaries selected for venous maturation (the main flow which was passing through one tube, now passes through another tube parallel to the previous one, while the previous one is squeezed. This amounts eventually to a sideways shift; see also Supplementary Movie 1 bottom left). PIV tracking of the displacement over a period of 7 h (Supplementary Movie 6) clearly shows that swelling between the arterioles and venules causes a physical, repelling interaction. This effect is accompanied by a “magnifying lens” (“Vasarely”) effect: capillary loops appear wide in the center and squeezed at the periphery (Fig. 3a, from Supplementary Movie 6). A shadowgraphic inspection (see Methods, Shadowgraph) of the surface (Fig. 3b) clearly shows a cascade of micro-swellings organized exactly like the vascular pattern. The tissue under the arteries forms bumps and veins explore the valleys between the bumps. These micro-swellings are witness to a complex distribution of compressive stresses, which is made visible on this quasi-2D tissue by imaging its topography. These stresses would be difficult to image in the bulk of a tissue.

### Delamination transforms in plane collaterals into vertical vessels

Tissue organization around the vessels can be clearly imaged by day 12 in the area where the CAM floats on the albumin, because the latter has a low optical density. Swollen cells along the arterioles are clearly seen to cross over the vessels in their proximal part (Fig. 3c). Distally the vessels are flattened, and cells cannot cross. The collaterals tend to follow the furrows between swollen cells. This explains why the plexus mesh has a gradient of diameter, with larger anastomoses closer to the arterioles. Such a gradient of plexus loops is observed ubiquitously (Fig. 3c, see also Fig. 2a). Also, the presence of swollen cells along arterioles explains the sawtooth structure of the tips of the arteries, with collaterals forming at regular intervals, as observed for example in Fig. 2a (arrows on the arterioles) and in Fig. 3c at a better resolution. These regularly spaced collaterals form regularly spaced chimneys by simply rotating vertically as vessel delaminates. (The transition from in plane sawtooth to 3D chimneys is caught in time-lapse in Supplementary Movies 1, 4, 5, and 8; careful inspection confirms that the positions of the vertical chimneys correspond to previous in plane collaterals).

### Tip flattening and tissue swelling form a repulsive interaction between arteries and veins

**Fig. 4:**
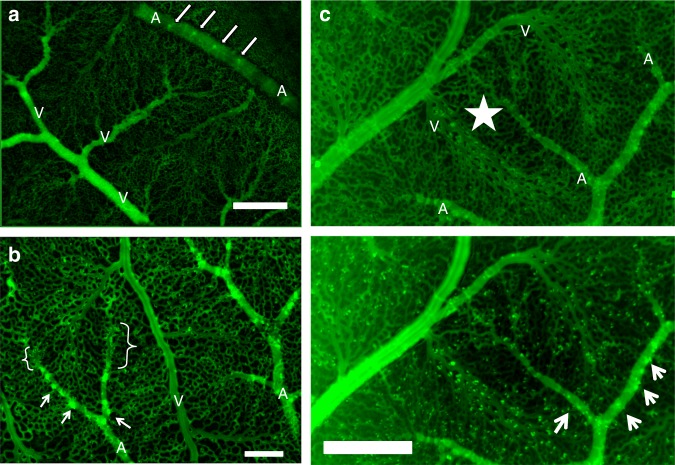
3D collaterals pointing upwards attract veins. The vertical chimneys along the arterioles serve as flow sources attracting hemodynamically the veins proximally. **a** Scale bar 300 μm. The figure shows a day 7 embryo, in which the vessels are quite coarse. It is already seen that vertical chimneys tend to “attract” the veins. **b** Scale bar 300 μm. The figure shows the vascular interlacing at day 10, it is observed that the capillary plexus is now oriented along the path starting at the vertical chimneys and flowing towards the presumptive vein. Careful inspection allows one to distinguish the swollen area under the distal flattened segment of the arteriole. The brackets show the flat distal segments. Around the two segments close to the brackets one sees a dimmer area corresponding to the flattened plexus. **c** Scale bar 250 μm. The panel shows typical interdigitating arteries and veins, in both “absorption” mode (Top), and in “average flux” mode (Bottom). The “Vasarely effect” is quite visible along the presumptive venous capillaries. One observes an area with lower flux (star) along the “flat” distal part of the vessel. The veins develop towards the vertical chimneys located more proximally along the arteries (arrows)

At day 7 (Fig. 4a), the forming venous tree is already oriented towards the vertical chimneys in the horseshoes formed by the branching arteries; they do not grow towards the tips of the arteries, although these are closer. By day 10, the capillary bed has adapted to the flow and the venous path swerves around the flat, terminal part of the arteriole (Fig. 4b). Extracting the average erythrocyte density (Fig. 4c), we find that blood flow is on average higher, proximally than at the tips of the arteries; flow is absent in the flat part of the arteries. The optical density is reduced in the swollen area, (see Fig. 4c: the star shows an area which appears darker because of the reduced number of capillaries and the reduced diameter of the remaining ones). Venules are oriented towards the vertical sources located proximally along the 3D segment of the arteries, and the venous path avoids the swollen area where the arteriole is flat.

**Fig. 5:**
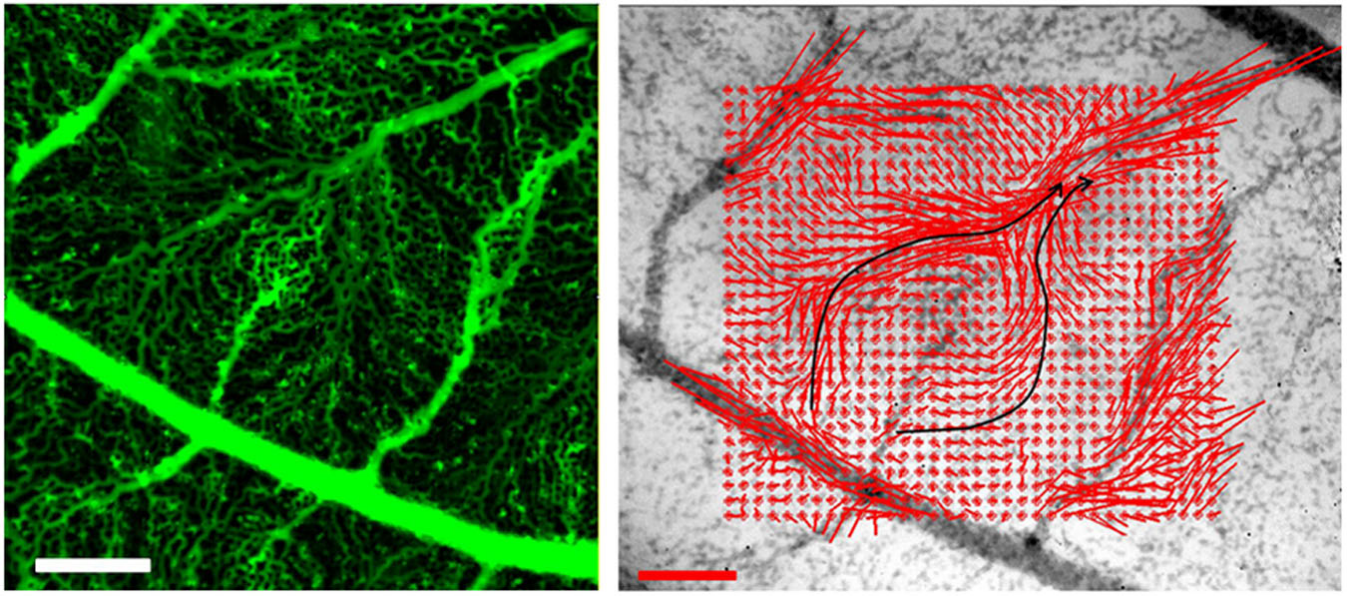
Measurement of the flow in a swollen area close to a growing arteriole. Left Scale bar, 250 μm. A growing arteriole is found, with veins visibly swerving around the tip of the arteriole. Right Scale bar, 200 μm s^−1^. The flow is imaged at 1000 fps, and the flow map is extracted by PIV, around the maximum of the heartbeat. The focus was done by eyeball in the plane of maximal speed. One observes a zone of exclusion; the flow springs from a more proximal part than the tip of the arteriole, it avoids the area surrounding the tip and reaches out towards the venule. The path of the flow is not directly from the apex of the arteriole to the venule

To confirm that the swelling had a direct impact on hemodynamics, we imaged the flow around distal swollen capillary beds with a Photron *FastCam* Camera, at 1000 frames per sec. (Supplementary Movie 7). We can generate both flow maps by PIV tracking of erythrocytes, and the image of the capillary bed with our algorithm (Fig. 5). We confirmed that blood flow swerves around the distal part of the forming arterioles and its surrounding capillaries. We stress that this observation is not incorporated in classical hemodynamic circulation models. If shear stress is the main cue for vessel growth^4–6^, this effect will induce a repelling interaction between arteries and veins. Veins will tend to grow *away* from the tip, towards more proximal areas.

### Insights from time-lapse observation

The temporal deformation of the vessels during CAM development can also be followed with our method (Supplementary Movie 1 obtained between days 10 and 13, Supplementary Movie 4 between days 7 and 8, Supplementary Movie 5 at day 6, Supplementary Movie 8 between day 10 and 12, Supplementary Movie 9 between 8 and 9). As arterioles progress, the tissue dilates conspicuously. This dilation is discontinuous: Supplementary Movie 9 shows accelerating “puffs” or “waves” of capillary formation. We also observed a rapid anisotropic extension of the arterioles after they delaminated (Supplementary Movies 5 and 8, by the end). Arterioles stuck to the ectodermal substrate resist elongational strain which likely inhibits their elongational growth.

## Discussion

**Fig. 6:**
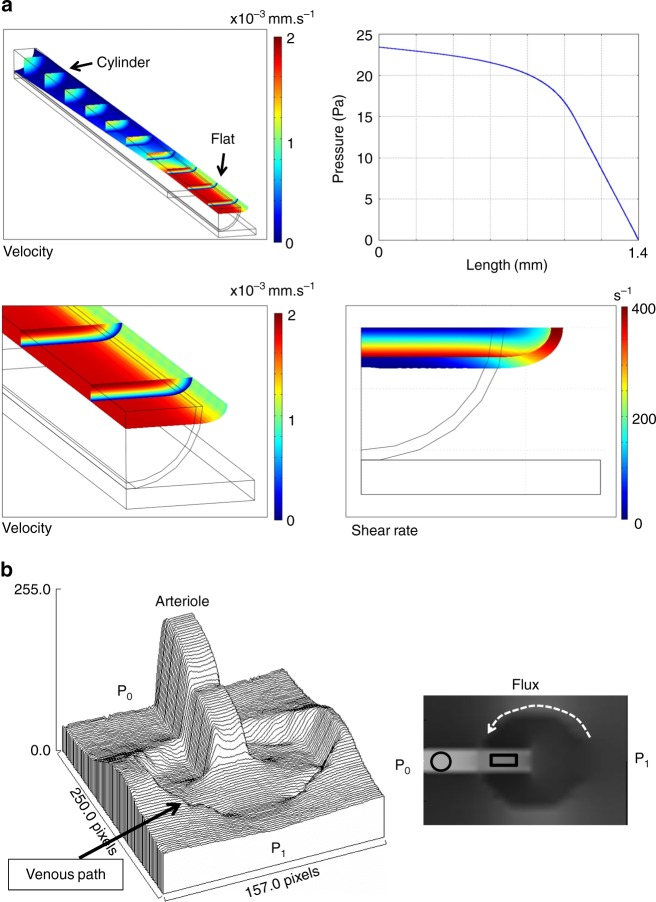
Poiseuille flow in a flattened tube. **a** We consider a cylindrical tube, becoming a flattened tube of identical perimeter (Top left, see Supplementary Movies 10 and 11). In this situation, the apparent width of the distal end of the flattened vessel is larger (by 42%), but the hemodynamic resistance is increased. We compute the pressure drop across the tube for a uniform flux, and indeed find a pressure drop larger in the flattened tube. The pressure profile is shown in (**a**) (top right): the hydrodynamic resistance at the flat exit is increased, here by a factor of ∼17. This shows that there is a quite strong increase of viscous drag. Since the shear is higher along the flattened tube, the flat tube will keep on spreading flat, if there is a developmental response to shear. The other plates show the flow velocity (bottom left) and shear (bottom right) in the tube. **b** The flux calculated around a growing tube whose distal part is exploring a flattened plexus where the hydrodynamic resistance is only 2.5 larger. The calculation finds an exclusion zone in the area of flattened capillaries where the flattened tube grows. An area of higher flux (dashed line) swerves around the distal flat part of the arteriole and flows towards the proximal 3D tube which is more conducting. The image to the left in **b** is the surface rendering of the calculated flux map to the right

Although the distal part of the arteriole looks wider, its hemodynamic resistance is increased because the vessel is flattened against the ectoderm (Fig. 6a). During flattening of a cylinder against a flat surface, the apparent diameter increases up to a width of πR, when the vessel is totally squeezed (Supplementary Movie 10). When flattening a tube from a circular cross-section (diameter D) to a roughly rectangular cross-section of apparent width 1.4D, the hydraulic resistance increases by a factor 17 (Fig. 6a, the model assumes a homogeneous fluid flowing in a smooth flattened pipe). Moreover, in the real system the tips of the vessels (arterioles or venules) explore a surrounding plexus of flattened capillaries which are in contact with the ectodermal surface too. The variation of blood viscosity with diameter (Fäerheus-Lindqvist effect^34^) could mitigate this effect by at most a factor 2 in the range of vessel diameters investigated here^34^. The flattened tip of the vessels therefore acts as a hemodynamic resistance.

The pressure increases upstream of the artery, until it is sufficient to induce delamination of the vessel and give it a circular cross-section. As from this point, the cylindrical vessel hangs beneath the ectoderm. 2D collaterals turn into 3D chimneys as the vessel sinks and cells come to lye over and press on them (Supplementary Movies 1, 4, 5, and 8). We numerically computed the hemodynamic pressure field and the fluxes around a growing arteriole whose tip is growing in a plexus pressed against the ectodermal surface, with a hydrodynamic resistance only 2.5 greater than the proximal part of the vessel. We find that the main flux goes around the tip of the arteriole and reaches out to the more proximal, 3D delaminated part (Fig. 6b). This explains why the flow swerves around the distal part and goes to the proximal regions, which are more 3D. This favors capillary remodeling into veins at a distance from the tips of arterioles. Veins will rather navigate towards older, more proximal, rounded-off parts of the arteries, where high flow in the vertical collaterals attracts veins hemodynamically.

**Fig. 7:**
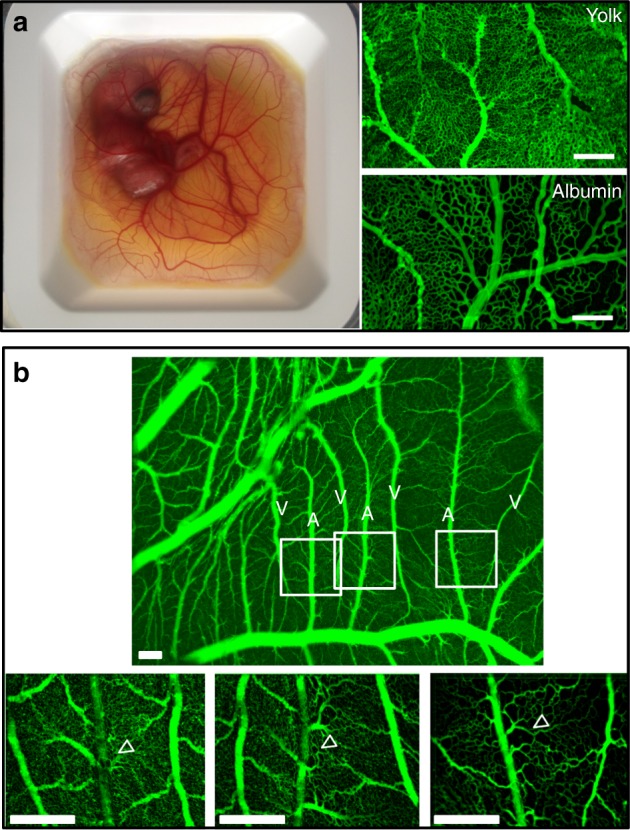
Gradient of vascular anastomoses. **a** Scale bar 200 μm. Image of an embryo in its plastic cup. Part of the CAM floats on the yolk (yellow area around the embryo), while part of the CAM floats on albumin (whiter area at the periphery). The capillary lattice found on the yolk at day 9 has a thinner mesh than the lattice found on the albumin. **b** Scale bar 250 μm. When the vasculature is well oriented, one can observe that, when passing from the area where the CAM floats on yolk (to the left) towards the area where the CAM floats on albumin (to the right), there is a monotonic increase in size of the vascular anastomoses along the forming arterioles (arrowheads). This contributes to the reported great variability in vascular patterns of the CAM, in addition to other known effects such as temperature or stretch inhomogeneities

This effect will certainly also depend on metabolic and biochemical stimuli. In a recent article, Clément et al.^35^ showed that the metabolic activity of the yolk-sac, via cell developmental pressure, explains the radial expansion and the morphogenesis of the first circular peripheral vein. The results presented here show the existence of micro-domains of swollen tissue at much smaller scale, and even at single-cell scale, that contribute to positioning each venule between the associated arterial horseshoe. These micro-domains are correlated to flat arterioles that adhere to the ectoderm. They are analogous to the early yolk-sac^35^, in that they correspond to domains that are locally ill-perfused. We have also observed that, in the same samples, and at the same developmental stage, there is a gradient of plexus anastomoses when going from the part of the CAM which is on top of the yolk towards the part of the CAM which is on top of the albumin. The loops are larger on the albumin (Fig. 7). Yolk is known to inhibit cell cycle^36^ while albumin is a more favorable medium for embryo development^27,28^. We hypothesize that cell metabolism and developmental dynamics may thereby modulate local compressive stresses exerted by the tissue on the forming vascular tree. In the same spirit as the peripheral vein expands radially even before the onset of perfusion, it is likely that the flat terminal segment of the arteries causes the surrounding tissue to swell and veins to be “pushed” away. On the contrary, proper perfusion tends to lower the stress. A possible rationale is that, in the presence of a small flow, metabolites accumulate in the tissue instead of being flushed away, thereby locally increasing the pressure.

What is the generality of these results and what is their impact on our understanding of vascular diseases? Generic physical phenomena should be observed in different contexts and taxons. We caution that heartbeat rate and erythrocyte size and shape are different in birds and mammals: spatial scales are not generic and may vary from one animal to the other. The observations and mechanistic explanations presented here rely on essentially three ingredients: a stratified tissue, a capillary plexus which adheres and creeps on a surface until it delaminates, and an increased pressure in ill-perfused areas which makes the tissue swell locally. The topological aspects linked to tissue stratification and surface creep of endothelial cells are universal. We cannot however exclude that the local pressure distribution depends genuinely on tissue type, its biological context (disease vs physiological) and the specific taxon. Especially, it is well known that ill-perfused tissue produces vascular endothelial growth factors (VEGF), which stimulate and guide capillary sprouting^37^. The density and orientation of capillary sprouting will certainly play a role in vascular morphogenesis and interlacing.

Our work predicts a possible general relationship between tissue stratification and vascular interlacing. Flattening of arteries onto cell layers is more likely in thin, flat tissues. This may explain why patterns obtained during de novo vascularization around 3D tumors show aberrant vessels which do not interlace properly (their vessel structure has been reported to be “unpredictable”^38^). If the stratification of the tissue is lost, as is often the case in tumor growth^39^, the mechanism presented here breaks down. Another prediction of the model relates the pressure in the vessels to the interlacing pattern: increased blood pressure will move the point of delamination further distally. This causes more distal interlacing with a greater probability of A-V shunts or fistulae. An A-V interlacing identical to that observed in the CAM is observed in the eye vasculature; hypertension causes vascular pathologies in which arteries and veines do not interlace properly^40^. A-V shunts and telengectasias are commonly observed in pathologies such as Rendu-Osler-Weber disease^41^.

As pointed out elegantly by Pries et al.^42^, the general problem of shunt formation is intrinsically asymmetrical: production of biomolecules transported by the flow in the vascular lumen may serve as biophysical information to prevent shunts downstream, but not upstream. The authors invoke the transfer of information by responses conducted along the vessel wall upstream, likely through gap junctions and ion channels (ref. ^42^ and references therein). This in turn requires another asymmetry to prevent aberrant downstream information conduction.

While biomolecular and metabolic feedback certainly plays a role in vascular patterning, we show here an additional A-V asymmetry related to the existence of a resistive segment at the tip of the arterioles. This hydrodynamic resistance varies dynamically during vascular morphogenesis and remodeling. This adds a physical layer to the problem of vasculature formation, which may contribute to explaining normal and pathological vascular trees.

While hemodynamics play an important role, one cannot deduce the construction plan of the vasculature from 2D measurements of the morphology or of the flow. Stresses exerted by surrounding tissues play an important role too: they modify the vessel cross section and determine the diameter of the initial capillary loops (although stresses are generally not directly visible, here, stresses could be imaged indirectly in the CAM by shadowgraph). Especially, parallel, interlaced vessels, as so often observed, obviously do not minimize viscous dissipation with respect to other geometries (parallel vessels double the dissipation with respect to direct A-V fistulae).

## Methods

### Embryos

Eggs were obtained at day 1 from EARL Morizeau, France. The embryos are incubated shell-less in a plastic cup. The plastic cup has a trapezoidal profile, which is important to image the CAM vessels by the edge, over a white background, esp. after day 10 (at this stage the CAM is so large that it reaches the edges of the cup). The eggs were opened and transferred to the plastic cup in a sterile hood. The shell was sterilized before being broken. The cup was placed in a large Petri dish (Duroplan 10 cms), with optical quality. 3 mm of PBS were poured around the plastic cup inside the Petri dish. The Petri dishes with the embryos were incubated in a Thermo Fisher incubator at 37 °C. For intra-vital imaging, the embryos, inside the Petri dish, were placed in between two heating stages (Minitüb gmbh, Ref. 12055/300). The top heating stage has a central round window. The central round window is covered with a copper plate 3 mm thick) with two slits, one slit for imaging, and one for shining light. The purpose of the copper plate is to avoid temperature gradients, which could generate gradients of growth rate, and also condensation.

### Ethics statement

These experiments are authorized by French law R214-87 modified by the Décret n°2013-118 to comply with European regulation.

### Optical imaging

We describe here the method to obtain an in vivo image of a live CAM with capillary resolution, after processing of primary images. An overview of data acquisition and processing for the measurement of vascular profiles is shown in Supplementary Figure 3. The primary images were acquired with a Stingray monochrome HD firewire camera from Allied Vision Technology (frame rate 15 Hz), or with a Basler CMOS monochrome HD USB camera (frame rate 42 Hz). The binocular was a Leica Macroscope F16 APO. The basic principle consists in averaging a movie. Supplementary Fig. 1 shows one typical plate in a movie of CAM imaging and the image obtained after processing the data. The basic tool is the Z-Project tool in the software Image J by which multiple images in a stack can be projected. In our case, we either *average* plates or generate the image containing *all minima* at each pixel. This amounts to extracting each point in the movie where at least one erythrocyte (black dot) has passed once during the time the movie plays. The most visible erythrocyte is kept for generating the image. As a result the “Minima-Image” shows the vasculature (the lumens), as if imaged by a homogeneous light from the inside, and the “Average-Image” shows the flows (the average red blood cell count passed at each point over the time), with the following caveat: static red cells give a bright spot (because of the permanent presence of a red cell at this spot). There is no need to inject a fluorescent dye.

However, if one would take a movie of the CAM, and do directly the “Average” or “Minima” image with the Z-Project tool in ImageJ as stated above, one would get a useless image. This is because there are ample movements in the embryonic tissue which ruin a naïve summation. The movements in the embryo are of 4 sorts. First: movements of the embryo itself, shaking and pulling all tissues chronically. Second: the heartbeat and vascular tone, which is cyclic and rapid; third: tissue contractions at long period and long wavelength (~minute); four: localized tissue contractions at short time periods (< minute). These contractions in the tissue were investigated specifically in more detail and their study will be presented elsewhere.

In order to get sharp images, one needs to realign (register) the plates in the stacks before averaging or extracting minima. We perform this registration with the StackReg plugin in ImageJ. However this is usually not sufficient either, because during the movements of the embryo, either the tissue is displaced too far away, or it is deformed in large proportions, or it moves in “Z” and gets blurred. This is why we developed two macros, one which discards blurred images, and one which discards images which are too different from a chosen reference image in the stack (a neat crispy one). The blurred images are discarded in the following way. Generally, blurred images arise from the oscillatory behavior of the heartbeat and of the embryo. With proper positioning of the objectives, the ROI will be acceptably focused for 50% of the time, and out-of-focus 50% of the time, due to the oscillation. We therefore need to discard approx. 1 out of 2 plates (whenever possible, we keep more). When a sharp feature gets out of focus, the light is diffused away, so that typically a sharp dark spot will become lighter in color (gray scale). We therefore select a crisp dark area in one sharp image, follow its gray level image-by-image and discard 50% of the images having a less sharp area, as deduced from measuring the gray level with the “measure” macro in ImageJ macro language. The initial reference feature is selected by the mouse. Therefore the operator just selects one sharp feature in one image of the stack, and the macro renders the 50% plates being sharp enough as compared to the reference. This step can be automatized for Time-Lapse.

Now, when all these steps are done before the registration of the files, one will generally still not get a useful image after Z-projection of the stack. This is because the image is essentially composed of erythrocytes, and these erythrocytes are flowing. The displacement of the erythrocytes amounts to a global average flow of a majority of the image (the red cells), which is enough to imply a slow backward drift of the image registration which strives to eliminate movements. This is why one last step must be performed (*patent pending*^30^): the plates in the movie are shuffled, in order to decorrelate the images of the red blood cells as much as possible. In practice, we acquire between 50 and 300 plates (at 15 hz) which amounts to 15–20 sec of video acquisition, and replace each 2K  + 1 plate between 1 and Nplates/2, by the plate found at Nplates/2 + 2k + 2. Reciprocally, we replace each plate found at Nplates/2 + 2k, by the plates 2k + 1 found between 1 and Nplates/2. This amounts to replacing each “next plate” by the farthest possible plate in the stack (modulo Nplates/2). By so doing, the plates are decorrelated, and the registration is not perturbed by erythrocyte flow. The plates can be unshuffled at the end to recover the registered flow. It is only after this registration, that we perform all steps explained above, and keep between 50 and 200 images, as final processable images prior to the Z-Project tool in Image J which performs the averaging or minima extraction. For some stacks, very difficult to process, a rapid visual analysis of the stack may be performed to discard manually anomalous images: indeed it may happen that the electronics generates spuriously one aberrant image in several hundreds, which suffices to ruin the algorithm (this was observed with the Basler camera). The final image can be color-flattened with the “Substract Background” tool, and rendered in either gray scale or a green look-up table, with adjusted levels (Adjust Brightness/contrast tool in ImageJ).

We also use a filter complementary to Red in order to enhance contrast (Leica green filter FS 505–550, associated to a Leica Lamp KL 1600 LED).

One last option, in order to get better images, consists in imaging vessels as far as possible from the embryo, especially after about 10 days of development, when the CAM reaches the edges of the plastic cup in which the embryos are incubated. In that area, the movements are generally of a smaller amplitude, and the vessels have a better contrast, being far from the embryo, and at some areas not even on the yolk sac.

This is how we get the crisper images of the capillaries (e.g. Supplementary Fig. 1).

The main drawback of the method is that it does not image dandling bonds, but only bonds in which there actually is a flow. The second drawback is the time: it may take up to 15 min to work out an image. The method is very time consuming if time-lapse movies are desired (e.g. Supplementary Movies 1, 5, and 8). The third drawback is that the method cannot image areas which undergo great deformations during the acquisition of the primary images (however, oscillatory displacements are processable).

However, the advantages of the method are that, first that it is extremely cheap; secondly, it does not require fluorescent labeling of cells, it does not require injection of fluorescent dies such as FITC dextran and it does not require fixation of the tissue; thirdly the method provides the magnitudes of the flow, at least qualitatively (calibration is currently under study). It can be implemented in time-lapse (see Supplementary Movies 1, 4, 5, and 6). It is also possible to overlap the actual flow on the vessel and follow red blood cells in the vasculature (see Supplementary Movie 12). Moreover, the method provides a volume rendering of the vascular surface profile, because the image is formed from absorbent particles dispersed inside the vessel lumen.

Finally, it should be noted that images are obtained by following individual erythrocytes passing over a homogeneous white field. Since the camera signal/noise ratio is optimal at higher level of light, and since the individual erythrocytes are each very small, the quality of the image obtained by integration is quite good. The resolution of the method is good enough to provide images of all capillaries at magnifications ×1 to ×9 with a binocular.

### Shadowgraph

The shadowgraph method consists in shining a parallel beam of light onto the surface. A parallel light is prepared by positioning a small source (fiber lamp Schott) at focal distance of a converging lens. A beam splitter positioned at 45° is used to have the beam descend vertically on the embryo surface. The light is reflected and the surface relief is observed optically with the binocular, and a CCD camera (Supplementary Fig. 2). Other examples of shadowgraphic imaging of embryonic surface can be found in ref. ^43^.

### Vessel profiles

We use optical absorption by erythrocytes to measure the vascular cross-section. The principle of the measurement is the following. First of all, the average erythrocyte flow cannot be used for measurement of the vessel cross-section, because it is well known that hematocrit segregation gathers erythrocytes flow in particular places of the vessels. In addition, that segregation depends strongly on vascular diameter^44^. Therefore, the average erythrocyte flow is not a direct measurement of the vessel profile. Instead, we use the “maximal absorption mode”. The principle is that statistically, at least one erythrocyte will explore any place in the vessel once, even a place which in average is less visited. Therefore if a long enough film is acquired, at least one erythrocyte has passed once at any spot. But if the movie is long enough, each vertical cross-section of the vessel will be filled at times by more than one red cell, rendering a larger absorption. And if the vessel is filled completely with red cells at least once in the recording time, the absorption is, in a crude approximation, proportional to the vessel thickness; therefore, the absorption level obtained by the “Minima” image, and for a long recording time, tends to approach the vessel cross-section. This turns into an optical absorption level proportional to vessel width (Supplementary Information Fig. 3). In principle, one should expect a blunting effect of light diffusion across the vessel. Also, in extracting the profiles, we assume that each vertical cross-section is filled completely with red cells at least once in the recording time, which may sound unrealistic. However, when performing the measurement on wide vessels (diam ~80 μm) which were obviously cylindrical, we were able to extract profiles which were well circular. If the analysis works for wide vessels, it should be even better for smaller vessels. Therefore, we assume that the blunting effects are negligible in the range of optical density, absorption and vessel thickness under study.

### Poiseuille flow in a flattened tube

We consider a tube of the circular cross-section. This cylinder is assumed to be compressed by contact with two symmetrical planes forming a wedge such that the cross-section of the cylinder varies from a cylinder, at the proximal end, down to a flattened cylinder. At the most distal part, the cylinder of initial diameter D is flattened such that the narrow direction has a gap D/4, and the wide direction an apparent width 1.42D. The deformation is calculated by the elasticity modules in Comsol (Supplementary Movie 9 shows the progressive deformation of 1/4th of the tube). In order to estimate the effect of such a deformation upon the flow of a fluid flowing in the tube, a numerical model has been developed in Comsol, based on Stokes equation solved by a finite elements method. Supplementary Movie 10 shows the variation of the magnitude of the flow, between the entry and the exit of the tube. The fluid is water (density 1000 kg/m^3^; dynamic viscosity 10^–3^ Pa.s). A flow rate has been chosen as 251,32 × 10^–14^ m^3^/s given a mean velocity of 0.5 mm/s or a centerline velocity of 1 mm/s. In the initial part of the tube, a Poiseuille flow takes place, the pressure gradient dp/dz is exactly −2500 Pa/m and the wall shear rate is exactly 50 s^−1^ (from Poiseuille formula). When the stream reaches the end of the tube the pressure gradient (absolute value) is increased by a factor of 17 (42500/2500). The wall shear rate is increased by a factor of 4 (200/50) along the curved part of the wall and of 8 (400/50) along the plane side.

The boundary conditions are: at the end of the tube the pressure is set to 0, at the entrance, a mean velocity is set to 0.5 mm/s. On the wall, a no-slip condition is imposed.

The finite element method needs to expand the solution onto a basis of polynomials. Lagrange polynomials of degree 2 for pressure and of degree 3 for velocity have been used leading to a stable numerical scheme and a good convergence.

All calculations have been performed by using the commercial numerical code “Comsol Multiphysics”

Results of computations:

The flow in the first part is a good test to benchmark the quality of the mesh and the accuracy of the expected values for pressure gradient and wall shear stress.

When the stream reaches the end of the tube the pressure gradient (absolute value) is increased by a factor of almost 18. The wall shear rate is increased by a factor of 4 along the curved part of the wall and of 8 along the plane side. Such results could predict an enhancement of stress and mechanotransduction upon the material of the wall.

### Flux around a flattened area

The distribution of flux for an arteriole (one proximal half “cylindrical”, one distal half “rectangular”) and growing across a partially flattened capillary plexus was obtained with the finite difference methods, implemented in python language. The conductivity *σ*[*i*,*j*] of the lattice is fixed as 0.4 in “flattened” capillaries, it is fixed as 1 (1 = 2.5 × 0.4) in cylindrical capillaries, as 4 in the flat part of the arteriole and as 10 ( = 4 × 2.5) in the cylindrical part of the arteriole. The scheme used is an explicit scheme with gradients discretized downstream. At each iteration the flux **J** = (*j*_*x*_,*j*_*y*_) is calculated as:

1 $${j_x}[{i},{j}] = \sigma [{i},{j}] \ast ({{V}}[{i},{j}] - {V}[{i} - 1,{j}])$$

2 $${j_y}[{i},{j}] = {\sigma }[{i},{j}] \ast ({V}[{i},{j}] - {V}[{i},{j} - 1])$$

The potential is calculated by solving iteratively in *k* the conservation law div(**J**) = 0, with an explicit scheme:

3 $${V}\left[ {{i},{j}} \right]_{k + 1} = {V}\left[ {{i},{j}} \right]_k + C \ast ({{j_x}}_k[{i},{j}] - {{j_x}_k[{i}} - 1,{j}] + {{j_y}}_k[{i},{{j}}] - {{j_y}}_k[{i},{{j}} - 1])$$

In which *C* is a constant chosen for convergence (in practice *C* = 0.05). The number of iterations for convergence is of order 100 000 for our calculations, in a matrix 26 × 41 pts.

### Code availability

The software for data analysis is available by the corresponding author upon reasonable request. The code may be used for reproducibility of the analyses present in the current study and it cannot be distributed. The algorithms of the code are protected by patent (Device for imaging blood vessels European Patent Office N° 18305795.9-1132 filed 22/06/2018).

## Electronic supplementary material

Supplementary Information

Description of Additional Supplementary Files

Supplementary Movie 1

Supplementary Movie 2

Supplementary Movie 3

Supplementary Movie 4

Supplementary Movie 5

Supplementary Movie 6

Supplementary Movie 7

Supplementary Movie 8

Supplementary Movie 9

Supplementary Movie 10

Supplementary Movie 11

Supplementary Movie 12

## Data Availability

Examples of the datasets generated during the current study, are available on the academic website of the author at: http://www.msc.univ-paris-diderot.fr/~vfleury/data-Nature-Comm-Biol-2018. Considering that each image in a T-L movie is obtained by processing about 300 HD (2 Mb) images, the size of the original data for 10 h of T-L on a 5 min interval basis is in the 75 Gb range. Complete datasets are available from the corresponding author on reasonable request.
