# Supplementary material for: Direct imaging of capillaries reveals the mechanism of arteriovenous interlacing in the chick chorioallantoic membrane
Source: Commun Biol. 2018 Dec 21;1:235. doi: 10.1038/s42003-018-0229-x (PMC6303259; doi:10.1038/s42003-018-0229-x)
Supplement: Supplementary file 1 — Supplementary Information [file 42003_2018_229_MOESM1_ESM.pdf]

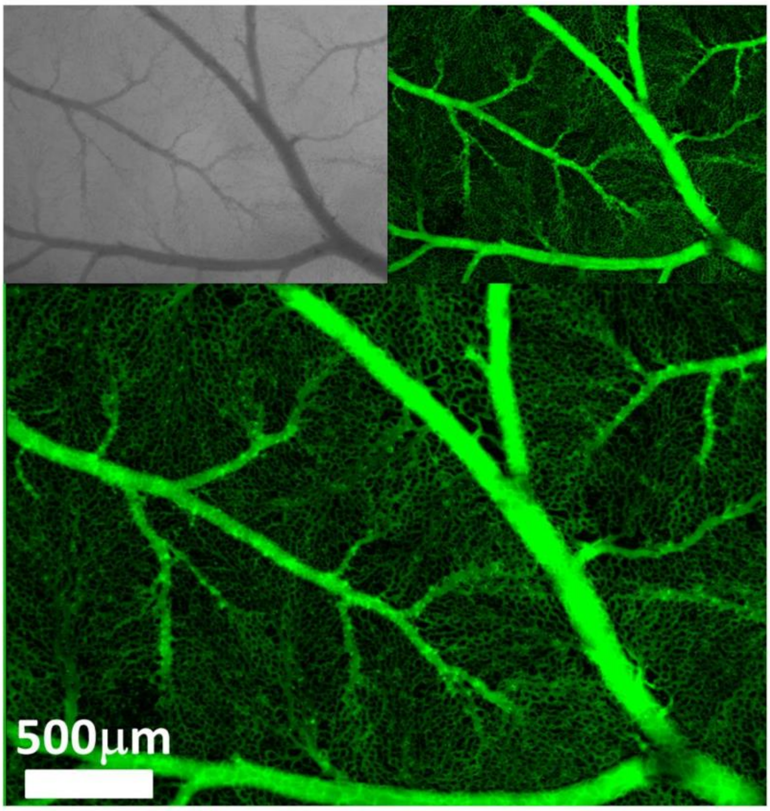

Supplementary Figure 1 Comparison of primary images and processed images. This figure shows one typical primary image in a HD movie (top left) and the final rendering after processing of the HD movie following the steps described in the main text. The capillary lattice appears in great detail, as "stained" by erythrocytes.

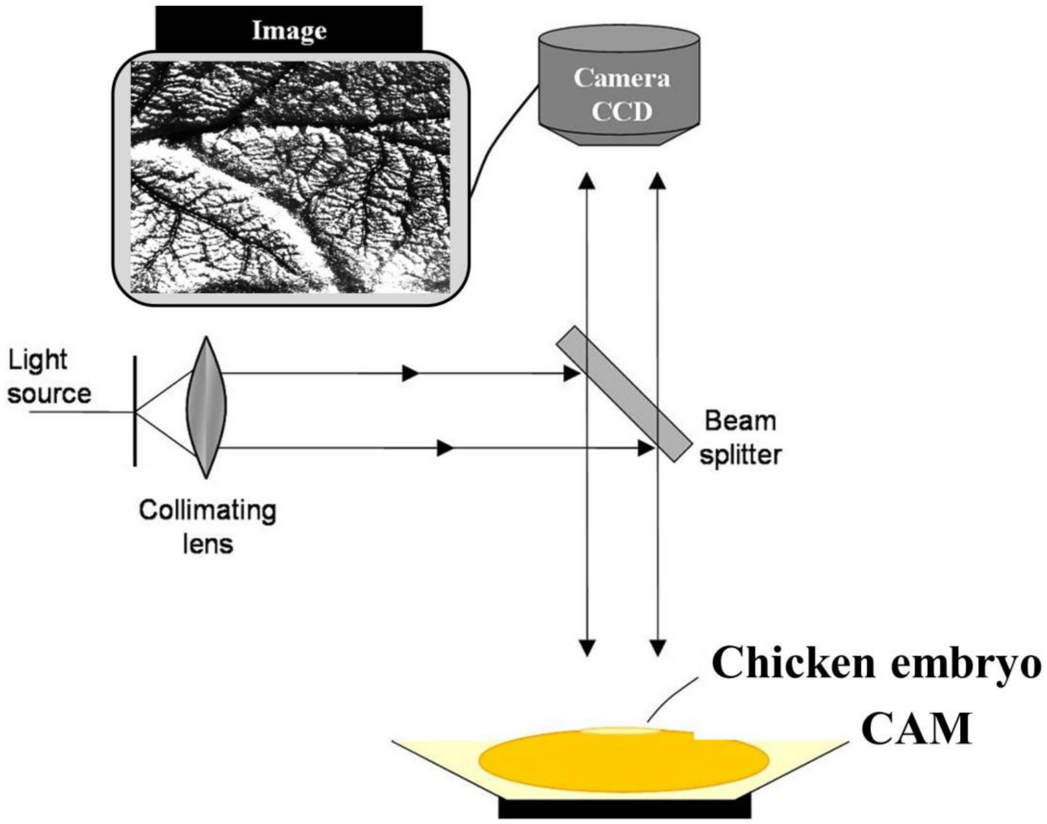

Supplementary Figure 2 Principle of the shadowgraphic imaging. A parallel beam of light is generated with a point source positioned at the focal distance of a lens. A 50% beam splitter is used to shine the beam vertically on the embryo, and film the specular reflection of the surface across the splitter. The surface relief scatters light away from the specular direction, thus revealing the valleys and bumps in the surface. The image shown in the schematic screen is an actual shadowgraph image of a day 12 CAM at magnification 1.6X.

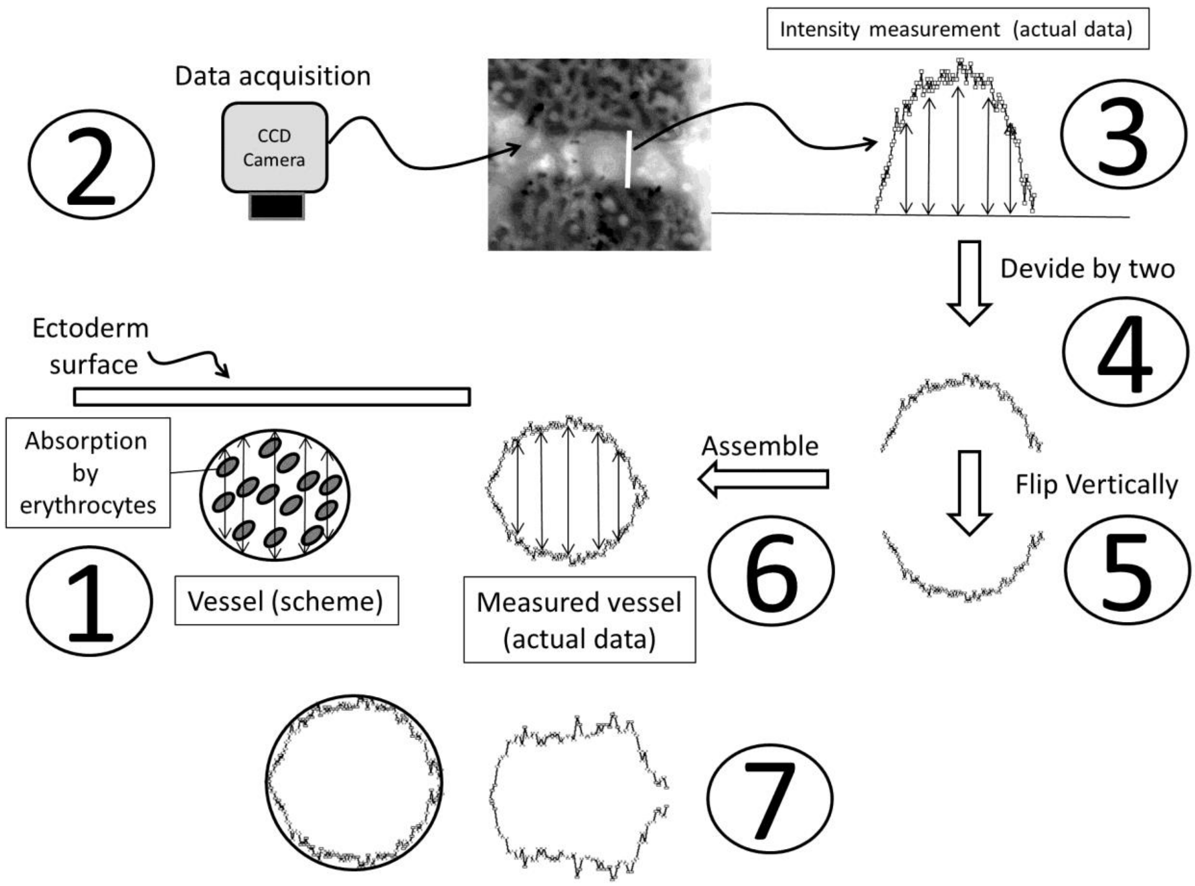

Supplementary Figure 3 Data acquisition and processing for measurement of vascular profiles. Given a vessel located in plane underneath a transparent ectoderm (1), the flow of erythrocytes is filmed for a long enough time (ex. 300 plates at 15Hz). A single image is formed by projection of the minima at each pixel in the stack of 300 plates (2). The image is inverted to get a gray level view of the absorption with 255=complete absorption. The absorption across the vessel in the vertical direction is in a crude approximation proportional to local vessel width (1). A profile of absorption perpendicularly to the vessel is extracted, at some straight location in between collaterals where a tubular segment is found (3). The profile of absorption has a height proportional to vessel width (3). The vessel can be reconstructed by symmetrizing (4,5) and assembling the two halves (6), if we assume a bilateral symmetry. Larger vessels which have higher pressure and are obviously almost cylindrical can be used for calibration, we found that the vascular cross section of cylindrical vessels is fairly circular (6,7), while distal vessels appear flat (7).

We assume in this set up that the optical refraction index of the plasma is identical to the optical refraction index of the tissue. This amounts to neglecting the cylindrical aberration due to the cylinder/plan diopter. For adjusted indices, the optical path across a cylinder embedded in a plate behaves as for a flat diopter, and there is no refractive distortion.
