# Supplementary material for: Direct imaging of capillaries reveals the mechanism of arteriovenous interlacing in the chick chorioallantoic membrane
Source: Commun Biol. 2018 Dec 21;1:235. doi: 10.1038/s42003-018-0229-x (PMC6303259; doi:10.1038/s42003-018-0229-x)
Supplement: Supplementary file 2 — Description of Additional Supplementary Files [file 42003_2018_229_MOESM2_ESM.docx]

**Description of Additional Supplementary Files**

**File Name**: Supplementary Movie 1

**Description**: **60 hours Time-Lapse of development of the CAM vasculature between day 10 and 13.** The video shows the transformation of capillaries into both arteries and veins. It shows the repulsive interaction between arteries and veins (for example in the bottom left corner, or in the middle-top). It shows the reduction of the apparent vascular diameter at the moment of delamination, and the transformation of collaterals into vertical chimneys. The data were acquired at Mag. 2X with primary frames 1920x1200 pixels. Due to ample embryo movements and overnight drift of the sample, a window of size 700x512 pixels could be extracted over the 60 hours of development (data available on a 5 min. time period). The stacks are processed automatically, it takes approx. 24 hrs Cpu to process the entire data on a laptop (a commercial version of the package will be available soon, patent pending^30^).

**File Name**: Supplementary Movie 2

**Description**: **Example of identification of the exact position of a delaminated vessel.** The flat part visible on top, is wider than the cylindrical part located underneath (Mag. 6X). The arrow in the final still image points to the exact position of the delamination.

**File Name**: Supplementary Movie 3

**Description**: **Registered data for arteries and veins at Mag. 4X, and day 8 of development.** The final still image is the corresponding processed image (we invert the gray scale to have the vessels appear bright).

**File Name**: Supplementary Movie 4

**Description**: **12 hours of development of the three small arterioles and the interdigitating arteries of Video 3, at Mag. 4X.**

**File Name**: Supplementary Movie 5

**Description**: **60 hours of development of the CAM between day 6 and 8 (Mag. 2.5X).** The videos shows many phenomena. We have circled in the still image at the end, one area where the swelling of a domain underneath an arteriole located centrally is quite conspicuous. Also in the top-right area, an arteriole undergoing a 2D to 3D transition, with formation of vertical chimneys from in plane sawtooth collaterals, is quite visible. The final longitudinal extension of the arterioles is visible by the end of the video (see for example top right arterioles).

**File Name**: Supplementary Movie 6

**Description**: **7 hours of swelling of a zone close to a flat arteriole (Mag. 3.2X).** It shows the swelling of the small anastomoses between the arteriole and the venule, and the squeeze of the venules between such swollen domains.

**File Name**: Supplementary Movie 7

**Description**: **High speed camera acquisition of the flow around a forming arteriole.** The flow was acquired at 1000fps, and reduced to 250fps for the animation. The flow swerves around the apex of the artery and a presumptive venous path forms which bypasses the tip of the artery. To the right, the vascular structure extracted by the algorithm (Ref. 30), from a video at 50fps (Average image).

**File Name**: Supplementary Movie 8

**Description**: **48 hours of development of the CAM between day 10 and 12 (Mag. 2.5X).** It shows the development of arterioles with the distal segment stuck to the ectoderm, the process of delamination of the arterioles, and rapid longitudinal extension of these arterioles after delamination.

**File Name**: Supplementary Movie 9

**Description**: **12 hours of development of an A-V interdigitation at Mag. 4X, around the moment when a new capillary wave occurs (day 8 embryo).** The increase in capillary density reduces the hemodynamic resistance, and a sudden flow increase occurs, which causes a brutal dilation of the vessels, and a more rapid morphogenesis. The Arterio-Venous repelling interaction is visible in the top-left corner, and also to the right.

**File Name**: Supplementary Movie 10

**Description**: **Flattening of a vessel at constant perimeter.** A circular vessel is assumed to be flattened at the bottom and top sides, by a flat slide. The video shows 1/4^th^ of the geometry (which is symmetrical with repect to the vertical and horizontal planes).

**File Name**: Supplementary Movie 11

**Description**: **Flow across the flattened tube from its cylindrical end towards the flat end.** The color scale gives the magnitude of the flow. The video shows 1/4^th^ of the geometry (which is symmetrical with repect to the vertical and horizontal planes).

**File Name**: Supplementary Movie 12

**Description**: **Zoom (1X=>6X) into the CAM structure.** The final part of the video shows the actual erythrocyte flow used to extract the vascular lumens, overlaid on the lumens. The green structure is the sum overtime of all maximal values of the points given by the circulating red cells.
